# Supplementary material for: A Critical View of the Application of the APEX Software (Aqueous Photochemistry of Environmentally-Occurring Xenobiotics) to Predict Photoreaction Kinetics in Surface Freshwaters
Source: Molecules. 2019 Dec 18;25(1):9. doi: 10.3390/molecules25010009 (PMC7017383; doi:10.3390/molecules25010009)
Supplement: Supplementary file 1 [file molecules-25-00009-s001.zip › 3.2.4_gcc-4.4.0.zip!/3.2.4_gcc-4.4.0/3.2.4_gcc-4.4.0/doc/octave/html/interpreter/Basics.html]

Basics - Untitled


Next: Sparse Linear Algebra,
Up: Sparse Matrices


---

### 21.1 The Creation and Manipulation of Sparse Matrices

The size of mathematical problems that can be treated at any particular
time is generally limited by the available computing resources. Both,
the speed of the computer and its available memory place limitation on
the problem size.

There are many classes of mathematical problems which give rise to
matrices, where a large number of the elements are zero. In this case
it makes sense to have a special matrix type to handle this class of
problems where only the non-zero elements of the matrix are
stored. Not only does this reduce the amount of memory to store the
matrix, but it also means that operations on this type of matrix can
take advantage of the a-priori knowledge of the positions of the
non-zero elements to accelerate their calculations.

A matrix type that stores only the non-zero elements is generally called
sparse. It is the purpose of this document to discuss the basics of the
storage and creation of sparse matrices and the fundamental operations
on them.

- Storage of Sparse Matrices- Creating Sparse Matrices- Information- Operators and Functions
